# Additional coronary sinus shocking lead as rescue therapy after multiple internal and external defibrillation failures

**DOI:** 10.1002/ccr3.968

**Published:** 2017-04-26

**Authors:** Samuel Chauveau, Arnaud Dulac, Laurent Sebbag, Elodie Morel, Philippe Chevalier

**Affiliations:** ^1^ Department of RhythmologyHospices civils de LyonLouis Pradel Cardiovascular HospitalLyonFrance; ^2^ Lyon Reference Center for inherited ArrhythmiasLouis Pradel Cardiovascular HospitalLyonFrance; ^3^ Heart Transplant DepartmentLouis Pradel Cardiovascular HospitalLyonFrance

**Keywords:** Coronary sinus, defibrillation failure, dilated cardiomyopathy, implantable cardioverter defibrillator

## Abstract

High defibrillation threshold (DFT) and defibrillation failure can lead to intractable ventricular arrhythmias. Additional coronary sinus coil is an effective strategy to achieve marked reduction in DFT. However, physicians should retain this might prevent future coronary sinus lead placement in case the patient would develop complete left bundle branch block.

A 52‐year‐old man was admitted for out‐of‐hospital cardiac arrest (OHCA) despite the presence of an implantable cardioverter defibrillator (ICD). Ventricular fibrillation (VF) was documented by emergency medical services. Sinus rhythm was restored after two external biphasic shocks at 200 J but three additional internal shocks were needed due to VF recurrence. On admission, ECG showed sinus rhythm and an incomplete left bundle branch block (Fig. 1).

**Figure 1:**
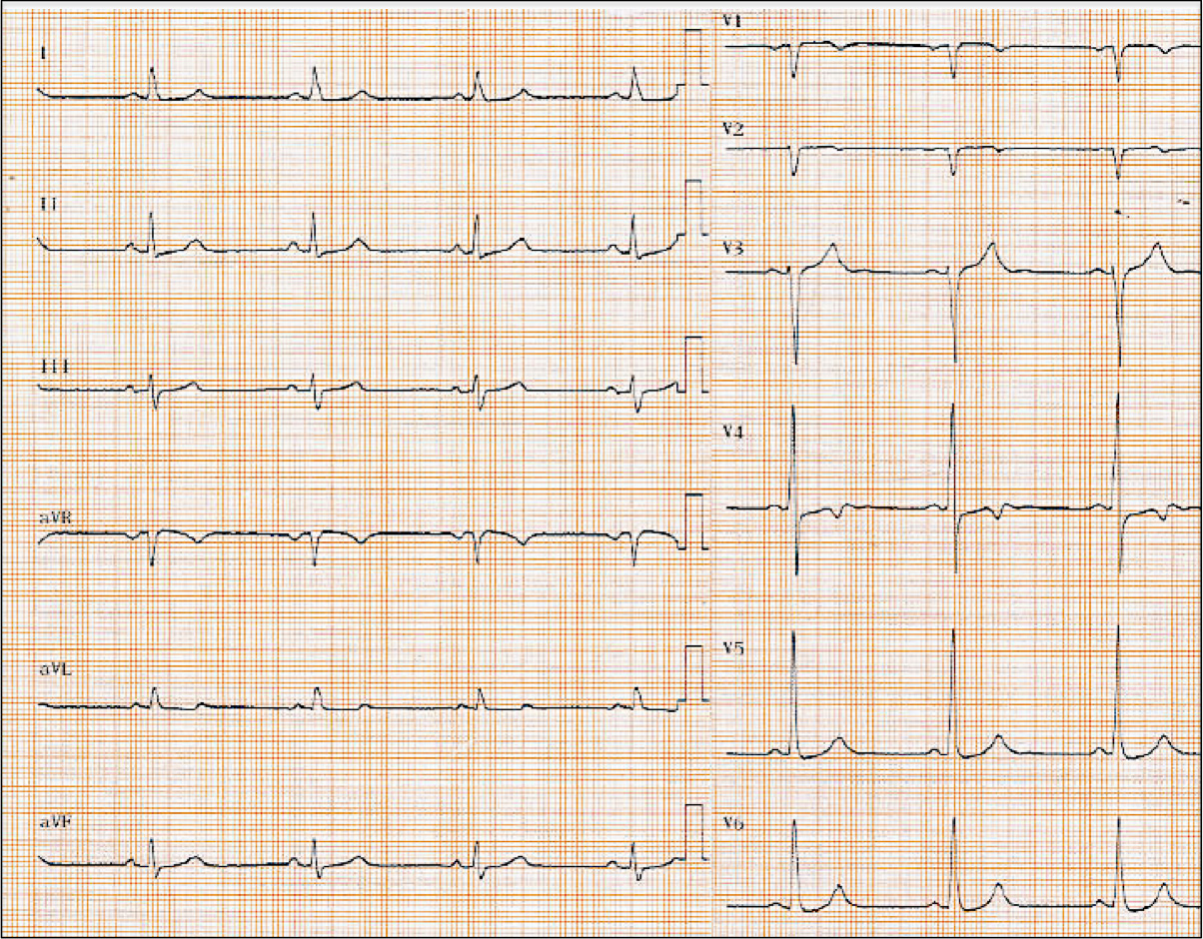
ECG on admission showed sinus rhythm and an incomplete left bundle branch block.

Three years earlier, the patient was diagnosed with inherited dilated cardiomyopathy (DCM) related to a mutation in the troponin T type 2 gene (TNNT2, p.Arg173Gln). The familial pedigree is shown in Figure 2A. Soon after being diagnosed with DCM and while under beta blocker therapy, ACE inhibitor, and spironolactone, he presented a first OHCA related to VF. After cardiac resuscitation and three external shocks at 200 J, sinus rhythm was restored. A left‐sided single‐chamber, high‐output ICD device (Boston Incepta, 41  J stored; 36  J delivered) using a dual‐coil DF‐4 lead positioned at the right ventricular (RV) apex was implanted (Fig. 2B). VF was induced by 50‐Hz burst pacing. The device delivered consecutively two shocks at 21 J and two shocks at 36 J, which failed to restore sinus rhythm. Sinus rhythm resumed after 10 external shocks and intravenous infusion of amiodarone. Lead parameters were in the normal range. Given multiple failures of internal and external defibrillation amiodarone at a daily dose of 200 mg was started.

**Figure 2:**
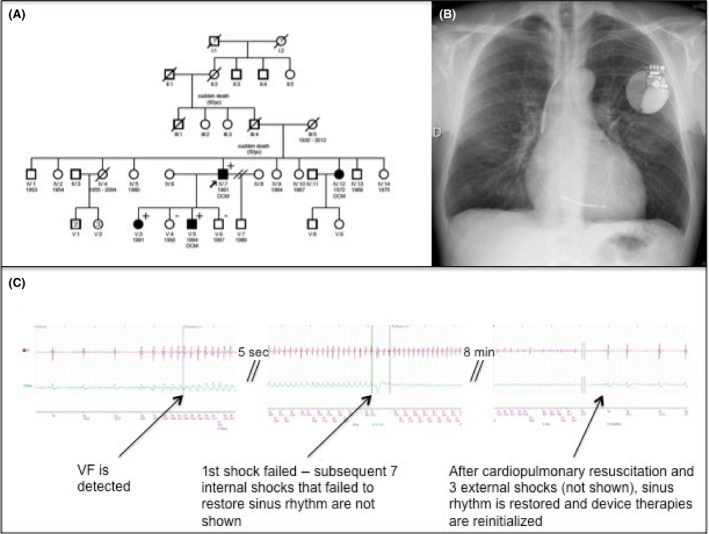
Panel A: familial pedigree; the patient is indicated with an arrow. Panel B: antero‐posterior chest X‐ray of the patient implanted with a dual‐coil defibrillator lead. Panel C: electrograms showing detected ventricular fibrillation and a first failed internal shock at 41 J (36 J delivered). After cardiopulmonary resuscitation and external shocks, sinus rhythm resumed. DCM = dilated cardiomyopathy; black‐filled circle or square = symptomatic male or female, respectively, for whom genetic testing has been performed; gene‐positive (p.Arg173Gln) or gene‐negative individuals are identified with a + or a −, respectively.

Analysis of stored electrograms showed that although VF (mean cycle length at 227 msec) was correctly detected by the ICD (Fig. 2C), eight high‐output reverse polarity shocks failed to restore sinus rhythm. After cardiopulmonary resuscitation and external shocks, sinus rhythm resumed (8 min after VF initiation, Fig. 2C) and device tachycardia therapies were reinitialized. Two recurrent VF episodes were detected and successfully terminated by three ICD shocks at 41 J. Shock lead impedance was normal between 43 and 48 ohms.

A high defibrillation threshold (DFT) caused by amiodarone treatment was considered, and amiodarone was replaced with sotalol (240 mg daily). After extraction of the dual‐coil lead, a single‐coil DF‐1 lead positioned in the RV apex and a 6937 Medtronic‐TRANSVENE‐SVC lead positioned in the coronary sinus were implanted. Given sinus bradycardia at 40 beats/min, an atrial lead was positioned into the right atrium to prevent RV pacing. Leads were connected to a high‐output ICD device (Medtronic Evera XT, 42 J stored). Chest X‐ray after ICD implantation is shown in Figure 3 (panels A and B). Testing was performed and VF was terminated on two consecutive occasions with a shock at 21 J (Fig. 3, panel C).

**Figure 3:**
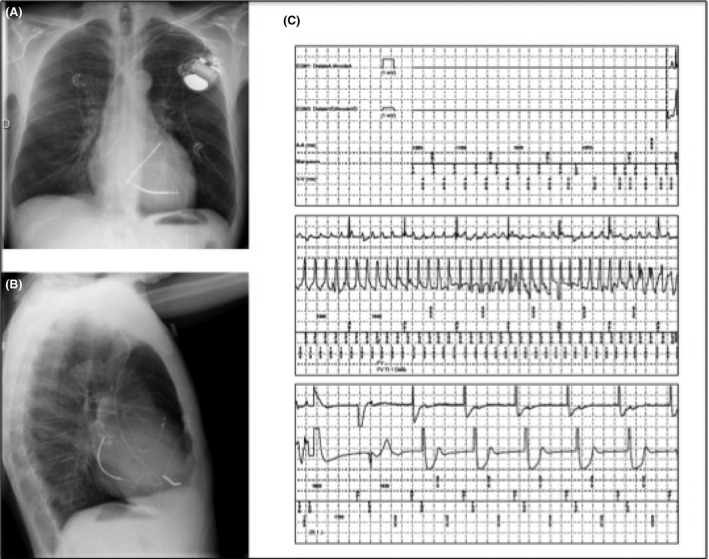
Panel A and B: antero‐posterior and right lateral chest X‐ray of the patient after removal of his initial defibrillation system and implantation of a single‐coil lead, a right atrial lead, and a coronary sinus coil. Panel C: defibrillation threshold testing showing VF induction by T‐wave shock converted to sinus rhythm after one internal shock at 21 J.

This case demonstrates the benefit of an additional left‐sided shocking lead in decreasing the DFT. As DF‐4 adaptors (high voltage splitter, Medtronic 5019) are not commercialized in France, the DF‐4 device and lead had to be replaced with a DF‐1 lead and device. However, it has to be kept in mind that this technique might prevent future left ventricle lead placement in case the patient would develop complete left bundle branch block. We also considered the use of an additional coil placed in the hemi‐azygos vein and the use a totally subcutaneous implantable defibrillator (S‐ICD). However, placing a coil in the hemi‐azygos vein remains challenging 1 and there is no data showing that S‐ICD improves the DFT safety margin when compared to ICD. The need of atrial pacing also precluded the use of a S‐ICD.

In studies of successful defibrillation with electrodes placed in the coronary sinus 2, 3, this configuration was used based on unacceptably high DFT. In this report, an additional coronary sinus lead was implanted for recurrent internal and external defibrillation failure of induced *and* spontaneous VF episodes. After implantation of a defibrillation floating lead into the coronary sinus, induced VF was terminated using 21 J. As spontaneous and induced ventricular fibrillation electrograms were not identical (VF minimum cycle length of 163 and 190 msec, respectively – Figs 2 and 3), it is likely but not certain that spontaneous VF would have been successfully treated. Finally, the respective role of sotalol and the coronary sinus shocking lead in decreasing DFT is uncertain 4, 5. Indeed, DFT testing was not performed in the presence of sotalol *before* ICD replacement.

In conclusion, concomitant use of sotalol and the addition of a left‐sided shocking coil lead are valuable strategies to achieve marked reduction in defibrillation threshold.

## Authorship

SC: wrote the draft and the revisions. AD, LS, ÉM: made corrections to the draft. PC: wrote the draft.

## Conflict of Interest

None declared.
